# A Standardized In Vitro Framework for Evaluating Hair Care Products: Hair Parameters and Efficacy Performance

**DOI:** 10.7759/cureus.110092

**Published:** 2026-06-02

**Authors:** Maheshvari N Patel, Nayan Patel

**Affiliations:** 1 Clinical Research, NovoBliss Research Private Limited, Ahmedabad, IND; 2 Pharmacology, Swaminarayan University, Ahmedabad, IND

**Keywords:** hair breakage, hair damage repair, hair frizziness, hair shaft, hair shine, in vitro, moisturizing, repair split ends, tensile strength

## Abstract

Background: There is an increasing demand for scientifically consistent methodologies to support the assessment of multifunctional cosmetic hair care claims while minimizing variability associated with in vitro testing. Therefore, the primary objective of this study was to develop a preliminary standardized in vitro human hair-tress-based framework for the comparative assessment of multiple cosmetic hair performance parameters under controlled laboratory conditions.

Materials and methods: This was a controlled in vitro standardization study conducted on standardized human hair tresses. Hair samples were randomly allocated into test and control groups. A structured damage-induction protocol was implemented to simulate real-world hair exposure, including chemical damage (surfactant washes), thermal damage (high-temperature styling), photodamage (ultraviolet A exposure), and environmental damage (pollution-related stress via ambient air exposure). Multiple evaluation methodologies were applied and standardized to assess hair sensory parameters, hair breakage, tensile strength, split-end repair/reduction, hair shine, hair volume, color retention, moisture retention, frizz control, and microscopic evaluation of hair shaft parameters. All assessments were performed using instrumental, image-based, and sensory techniques under controlled laboratory conditions.

Results: Application of the test product to standardized hair tresses resulted in measurable changes across several evaluated hair parameters, showing improvement in hair breakage count by 21.00 ± 6.07 counts, hair tensile strength by 25437.01 ± 6169.93 gm/cm², hair shine by 2.15 ± 0.30, hair volume by 499.66 ± 34.30 cm², and reduction in hair frizziness by 89.20 ± 5.93 cm². In contrast, the control group did not demonstrate any notable improvement in the assessed parameters following the application procedure. Hair breakage increased to 51.50 ± 10.95, hair tensile strength slightly decreased to 23468.01 ± 7519.68, hair shine reduced to 1.40 ± 0.17, hair volume was reduced to 423.16 ± 15.81, and hair frizziness increased to 133.62 ± 8.56.

Conclusions: The present study proposes a preliminary integrated in vitro hair-tress-based framework for evaluating the multifunctional performance of cosmetic hair under controlled laboratory conditions. The methodology demonstrated measurable changes in multiple hair parameters following product application, supporting its potential utility for comparative cosmetic hair assessment and claim support.

## Introduction

Hair studies across the globe have experienced considerable growth recently, reflected in increased consumer demand for a multifunctional range of hair products formulated to improve various aspects of hair health and appearance. These products frequently claim benefits such as strong hair, long-lasting shine, and no frizz, which can be assessed using standardized methodologies. Supporting such cosmetic claims requires scientifically consistent testing methodologies that objectively evaluate hair fiber performance under controlled laboratory conditions [1].

Human hair is a complex biological fiber primarily composed of keratin proteins arranged in hierarchical structural layers within the individual hair shaft, namely the cuticle, cortex, and medulla. These structural layers determine important hair properties such as shine, elasticity, strength, and resistance to environmental damage. Daily environmental exposures such as surfactants, ultraviolet radiation, heat styling, and pollution can disrupt the cuticle structure, resulting in disrupted porosity, mechanical fragility, hair breakage, and frizz formation [2].

The in vitro hair-tress framework is widely used to assess the efficacy of hair care products under controlled laboratory conditions. Standardized hair tresses provide an experimental substrate for assessing multiple hair parameters. These frameworks are particularly useful for studying the effects of formulations on hair integrity and performance while minimizing variability associated with in vivo testing [3].

Various instrumental and image-based analytical techniques have been employed to quantify changes in hair properties following hair damage. Methods such as hair colorimetry, hair shine, strength, and image analysis are commonly used to evaluate parameters [4]. Additionally, sensory evaluation scales are frequently incorporated to assess experts’ evaluation attributes, such as wet and dry combability, hair bounce, hair frizz, smoothness, hair shine, hair volume, hair moisture, conditioning effect, and overall manageability of hair fibers.

However, despite the availability of assessment techniques, the major problem in hair claim substantiation remains the lack of harmonized and standardized testing frameworks that integrate multiple hair evaluation methodologies into a single experimental system. Variability and lack of standardization in experimental procedures, damage-induction models, environmental conditions, and measurement tools and scoring scales can lead to inconsistencies in results across studies, thereby limiting the reliability of product claims.

Therefore, there is a growing need to establish scientifically consistent in vitro frameworks to evaluate multiple cosmetic hair parameters under controlled laboratory conditions. Such frameworks may help improve methodological consistency, reduce experimental variability, and support comparative assessment of cosmetic hair performance.

The primary objective of the study was to develop a standardized in vitro human hair tress framework for the comparative assessment of cosmetic hair fiber performance parameters under controlled laboratory conditions. Secondary objectives were to describe the application of multiple instrumental, image-based, and sensory methodologies to evaluate mechanical, optical, and structural hair properties following standardized damage-induction procedures. We hypothesized that integrating standardized mechanical, optical, sensory, and image-based methodologies into a single in vitro framework could improve methodological consistency and reduce variability in assessing cosmetic hair performance.

In vitro testing using standardized hair tresses is widely accepted in hair care research as a reliable, reproducible approach for evaluating the performance of hair care formulations under controlled laboratory conditions. These models allow precise control over experimental variables such as damage induction, product application, and environmental exposure, thereby reducing variability. Moreover, in vitro hair tress models enable direct assessment of mechanical, optical, and structural properties of hair fibers, including tensile strength, breakage, shine, and surface morphology, which are critical for substantiating claims. Previous studies have demonstrated the suitability of hair tress models for evaluating the effects of treatments and environmental stressors on hair fiber integrity and performance, supporting their use as standardized tools in in vitro assessments [5].

## Materials and methods

Study design

This study was conducted as a controlled in vitro study to assess experimental methodologies for evaluating multifunctional hair care claims using hair tresses. The experimental framework was designed to develop standardized testing procedures to evaluate multiple hair care parameters, including hair sensory characteristics, split-end repair, hair breakage, shine, tensile strength, color retention, volume, frizz control, moisture retention, and structural hair parameters. All evaluations were conducted under controlled laboratory conditions to ensure methodological consistency and minimize variability.

Hair Tress Sample Specifications

For the experimental procedure, standardized hair tresses were used. Hair tresses used in the study were commercially procured from Gujarat Hair Studio. A total of 12 black hair tresses were used and randomly assigned into two study groups: the test group (n = 6 hair tresses) and the control group (n = 6 hair tresses). For the evaluation of split-end repair, 12 separate hair strands exhibiting split ends were selected, with six strands assigned to each study arm. Similarly, 12 bleached hair tresses were used to assess hair color retention.

To ensure consistency across experiments and minimize procedural variability, all hair tresses were standardized prior to the study's initiation. It was ensured that only virgin human hair, free from chemical treatments such as bleaching, coloring, and straightening, was included in the study. The hair samples were selected from uniform, naturally black, straight hair, and each hair tress was 30 cm long and weighed 7-8 g. Additionally, all hair tresses were visually checked before inclusion in the study to confirm the absence of any visible structural deformities and to ensure uniform baseline characteristics across all hair tress samples.

Randomization and Blinding

Hair tresses were randomly assigned to either the test or the control group using a randomization schedule. Each tress was labeled with a unique identification code to ensure unbiased allocation to any of the groups. To minimize assessment bias, the study was conducted in a blinded manner. Personnel involved in instrumental measurements, image capturing, and analysis remained blinded to product allocation until completion of all assessments.

Damage Induction Procedures

To mimic realistic hair-damage conditions, the damage induction process was performed prior to product application. This procedure included multiple stages designed to replicate environmental and related factors that contribute to hair damage. First, chemical damage was induced by subjecting the hair tresses to 10 consecutive washes using a 14% sodium lauryl ether sulfate solution. Following this, thermal damage was simulated using a hair straightener set to 230°C, with 100 repeated vertical downward strokes applied to the hair fibers. Each stroke was maintained for approximately 20 seconds to mimic routine heat styling. Additionally, photodamage was induced by exposing the hair tresses to ultraviolet A (UVA) radiation using a UVP UVL-21 lamp (4 W / 365 nm; P/N 95-0018-03, 0.16 A / 230 V~50/60Hz). Hair tresses were exposed to UVA radiation at 365 nm for 30 minutes at a fixed distance from the UV source under controlled laboratory conditions. Finally, to replicate the effects of environmental stressors, the hair tresses were exposed to ambient conditions at 30°C and 40% humidity for 48 hours. The AQI level was recorded as 69%. Environmental exposure conditions included particulate matter and gaseous pollutants, including PM₂.₅ (20 µg/m³), PM₁₀ (26 µg/m³), carbon monoxide (CO: 220 ppb), sulfur dioxide (SO₂: 10 ppb), nitrogen dioxide (NO₂: 13 ppb), and ozone (O₃: 29 ppb). These combined procedures enabled the generation of damaged hair samples for subsequent evaluation of the test product.

Product Application

Hair tresses assigned to the test product group were treated with Neela Bhringadi Hair Oil, while control samples received tap water. The major ingredients of Neela Bhringadi Hair Oil include Nili Rasa, Bhṛingaraja Rasa, Satakratu Lata Rasa, Dhatri Phala Rasa, Aja Ksira, Narikela Ksira, Mahisi Ksira, Dhenudugdha, Tila Taila (Murcchita), Yaṣṭimadhu, Gunja, and Anjana (Rasanjana).

Approximately 3 mL of the test product was uniformly applied along the entire length of the hair tresses to ensure consistent coverage. The product was left on the hair tresses for two hours before washing with Clinique Plus shampoo using normal water at approximately 30°C. Each application-wash cycle was performed with a 24-hour interval between consecutive cycles. This procedure was repeated for a total of 12 application-wash cycles under controlled laboratory conditions.

Instrumental Details

Multiple instruments were employed to assess various hair parameters, including the Testronix Tensile Tester for tensile strength, Image-Pro software for frizz analysis, Glossymeter GL 200 for hair shine, Colorimeter CL 400 for color assessment, and Magnus Microscope for split-end evaluation, each supported by respective software systems to ensure accurate and reproducible measurements (Table 1).

**Table 1:** Instrumental details

Instruments	Manufacturing details	Software details	Applications in the study	Manufacturer name and location
Testronix Tensile Tester	Testronix	2	Measure the tensile strength of the hair	Testronix Instruments; I-10A, Dlf Industrial Area, Phase-I, Near Nhpc Metro Station, Faridabad, Haryana, 121003, India
Image-Pro Analysis	Media Cybernetics	Image-Pro, 10.0.13	Image-Pro is a comprehensive application for evaluating hair frizziness	Media Cybernetics, Inc.; 1700 Rockville Pike, Suite 240, Rockville, Maryland, USA 20852
Glossymeter GL 200	Courage + Khazaka Electronic GmbH (C+K)	MPA CTplus (1.1.6.5)	Measure the hair gloss	Courage + Khazaka Electronic GmbH; Mathias-Brüggen-Str. 9150829 Köln, Germany
Colorimeter CL 400	Courage + Khazaka Electronic GmbH (C+K)	MPA CTplus (1.1.6.8)	Measure the change in hair color	Courage + Khazaka Electronic GmbH; Mathias-Brüggen-Str. 9150829 Köln, Germany
Magnus Microscope	Magvision Analytics Magvison	X64, 4.11.22728.20230606	Hair split-ends	Magnus Opto Systems India Pvt.; A-3, Sector-81, Phase-II, Noida-201305, Uttar Pradesh, India

Evaluation parameters

Multiple evaluation methodologies were standardized as part of the in vitro testing framework.

Hair Sensory Assessments

Hair sensory assessments were conducted by three trained dermatologists using predefined dermatological scoring scales.

Hair Breakage

Hair breakage was assessed using wet and dry fatigue testing with the Testronix Tensile Tester in wet and dry hair, respectively. Broken hair fibers were counted after standardized combing strokes.

Hair Tensile Strength

Hair tensile strength was measured using a mechanical tensile strength tester. Individual hair fibers were mounted between standardized clamping points at a fixed clamping distance of 0.8 cm and tested for force until breakage; parameters such as breaking force and elongation at break were recorded.

Split-End Analysis

Split ends were evaluated by microscopic examination with a biological microscope at 4× magnification, and images were captured and analyzed using Image-Pro software.

Hair Shine Measurement

Hair shine was measured using a Glossymeter GL 200 based on reflected light intensity from the hair tress surface under controlled environmental conditions (temperature: 5-40°C; relative humidity: 30-70% RH) in a closed, dark room to minimize external light interference. Measurements were recorded from the rear end/tip region of the hair tresses, with up to 10 readings obtained per hair tress at each time point for standardized assessment.

Hair Frizz Analysis

High-resolution digital images of flyaway hairs were captured from multiple orientations. Image analysis software was used to quantify hair tress area and flyaway regions to evaluate hair frizz control.

Hair Color Retention

Hair color parameters were measured using Colorimeter CL 400 based on the L*, a*, and b* color space system.

Moisture Retention and Porosity

Hair hydration characteristics were evaluated using gravimetric analysis and a floating porosity test to assess water uptake and retention properties and hair porosity assessment.

Statistical analysis

Continuous variables were summarized using descriptive statistics, including the number of observations (N), mean, standard deviation (SD), median, minimum, and maximum values, as well as the mean change from baseline (CFB) and the percentage change from baseline (%CFB). Categorical variables were presented as frequencies and percentages, and graphical representations were used where appropriate. Normality of all parameters was assessed using the Shapiro-Wilk test.

For inferential analysis, paired t-tests were applied to assess changes from baseline for hair breakage, hair tensile strength, hair volume, split-end analysis, length of first and second strands, hair frizziness, hair shaft diameter, water uptake, and water retention parameters. L*, a*, and b* color parameters were analyzed using repeated-measures ANOVA. Mauchly’s test was used to assess the assumption of sphericity, and when this assumption was violated, Greenhouse-Geisser corrections were applied. Post hoc pairwise comparisons between visits were adjusted using Bonferroni correction.

Hair shine parameters were analyzed using the Wilcoxon signed-rank test. All statistical analyses were performed using SPSS Statistics version 29.0 (IBM Corp. Released 2022. IBM SPSS Statistics for Windows, Version 29.0. Armonk, NY: IBM Corp.).

## Results

A total of 12 hair tresses were included in the study. Out of these, six (50.00%) hair tresses were treated with the test product, and six (50.00%) hair tresses were treated with the control product.

Hair sensory evaluations

**Figure 1:**
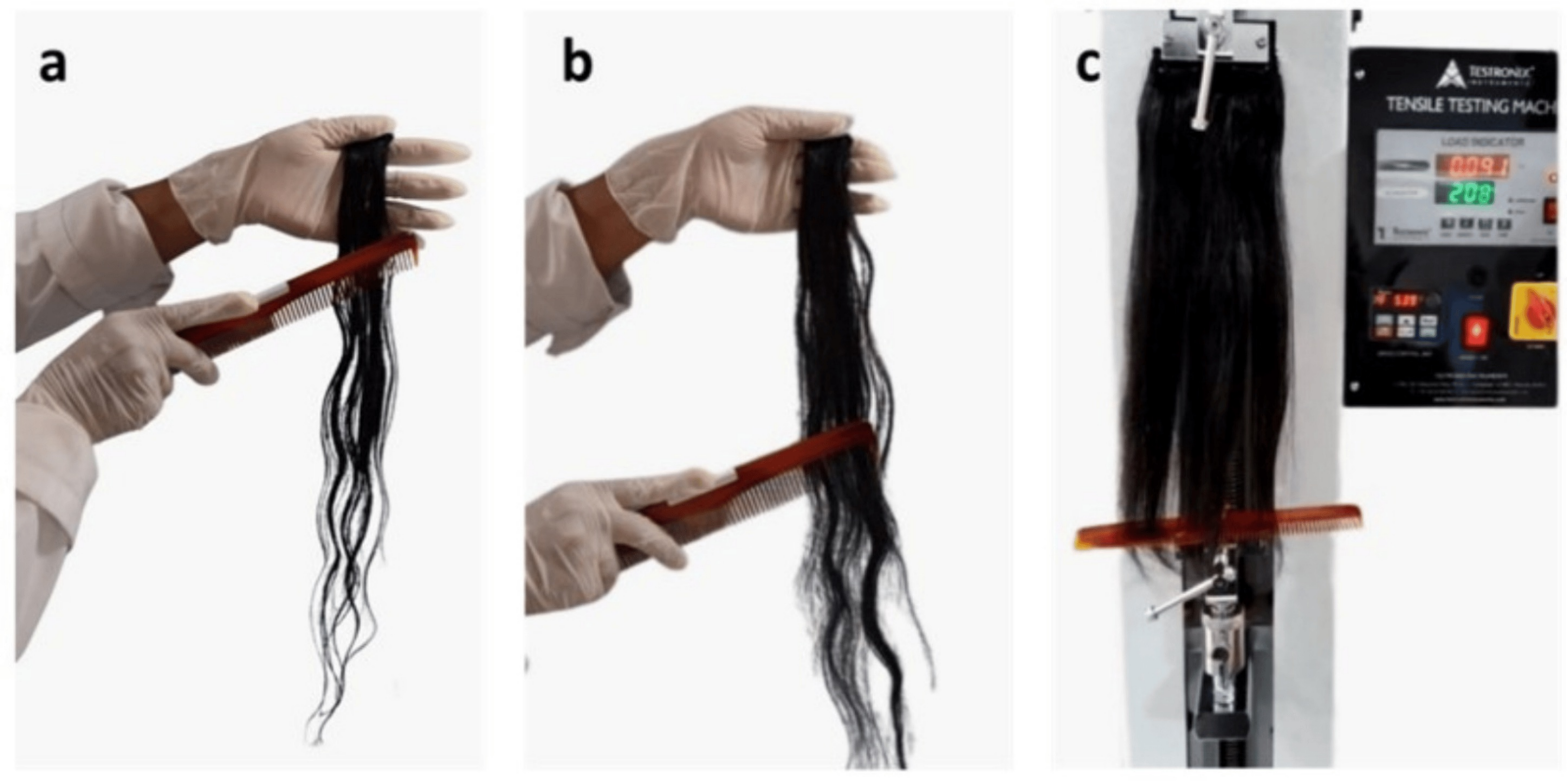
Combability assessment Representative images illustrating the procedure for assessing hair breakage under standardized conditions. (a) Manual combing of the hair tress under wet conditions for breakage evaluation; (b) manual combing under dry conditions to assess hair breakage; and (c) instrumental evaluation of hair breakage using the TESTRONIX Tensile Tester.

**Figure 2:**
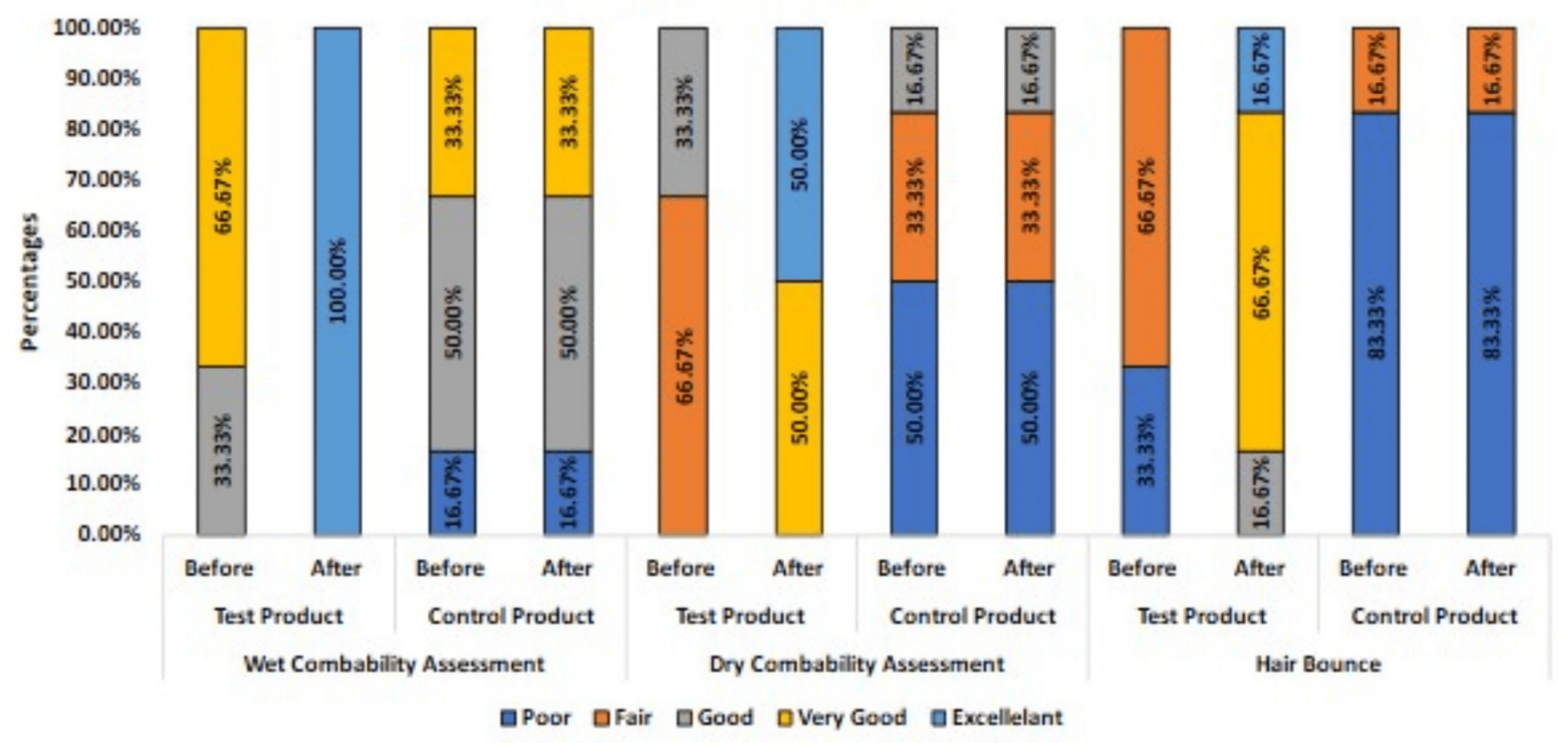
Sensory assessment evaluation Wet combability, dry combability, and hair bounce were assessed by a trained dermatologist.

Following the application of the test and control products to standardized hair tresses, a noticeable improvement was observed in the hair sensory evaluation parameters in the test product arm. The test-treated hair tresses demonstrated a positive shift across multiple sensory attributes, including wet and dry combability, smoothness, shine, frizz control, and overall manageability. In contrast, the control arm exhibited only minimal or negligible changes in these parameters following the application procedure, indicating limited impact under the same experimental conditions (Figures 1-2, Table 2).

**Table 2:** Change in hair sensory parameters from before application to after application Distribution of hair sensory evaluation parameters before and after application of the test and control products on standardized hair tresses. Data are presented as the number of hair tresses (count, n) with corresponding percentages. Statistical analysis was performed using a count percentage to assess changes from baseline within each group.

Statistical parameters	Test product, counts (percentage)		Control product, counts (percentage)	
	Before application	After application	Before application	After application
Hair shine assessment				
Very dull	0 (0%)	0 (0%)	1 (16.67%)	1 (16.67%)
Dull	4 (66.67%)	0 (0%)	5 (83.33%)	5 (83.33%)
Slightly shiny	2 (33.33%)	0 (0%)	0 (0%)	0 (0%)
Moderately shiny	0 (0%)	4 (66.67%)	0 (0%)	0 (0%)
Very shiny	0 (0%)	2 (33.33%)	0 (0%)	0 (0%)
Hair frizz assessment				
None	0 (0%)	1 (16.67%)	0 (0%)	0 (0%)
Low	0 (0%)	5 (83.33%)	0 (0%)	0 (0%)
Moderate	3 (50.00%)	0 (0%)	2 (33.33%)	2 (33.33%)
High	3 (50.00%)	0 (0%)	3 (50.00%)	3 (50.00%)
Very high	0 (0%)	0 (0%)	1 (16.67%)	1 (16.67%)
Hair smoothness assessment				
Very rough	1 (16.67%)	0 (0%)	1 (16.67%)	1 (16.67%)
Rough	5 (83.33%)	0 (0%)	5 (83.33%)	5 (83.33%)
Smooth	0 (0%)	0 (0%)	0 (0%)	0 (0%)
Moderately smooth	0 (0%)	4 (66.67%)	0 (0%)	0 (0%)
Very smooth	0 (0%)	2 (33.33%)	0 (0%)	0 (0%)
Hair volume assessment				
Greatly decreased	0 (0%)	0 (0%)	0 (0%)	0 (0%)
Moderately decreased	2 (33.33%)	0 (0%)	1 (16.67%)	0 (0%)
Slightly decreased	4 (66.67%)	0 (0%)	5 (83.33%)	0 (0%)
Unchanged	0 (0%)	0 (0%)	0 (0%)	6 (100%)
Slightly increased	0 (0%)	4 (66.67%)	0 (0%)	0 (0%)
Moderately increased	0 (0%)	2 (33.33%)	0 (0%)	0 (0%)
Greatly increased	0 (0%)	0 (0%)	0 (0%)	0 (0%)
Hair moisture assessment				
Very dry	3 (50.00%)	0 (0%)	4 (66.67%)	4 (66.67%)
Dry	3 (50.00%)	0 (0%)	2 (33.33%)	2 (33.33%)
Moderately dry	0 (0%)	0 (0%)	0 (0%)	0 (0%)
Moisturized	0 (0%)	4 (66.67%)	0 (0%)	0 (0%)
Very moisturized	0 (0%)	2 (33.33%)	0 (0%)	0 (0%)
Hair porosity assessment				
Low porosity	6 (100%)	1 (16.67%)	6 (100%)	6 (100%)
Normal porosity	0 (0%)	5 (83.33%)	0 (0%)	0 (0%)
High porosity	0 (0%)	0 (0%)	0 (0%)	0 (0%)

Hair breakage assessment

Following the application of the test and control products, the test product arm showed reduced hair breakage compared to the baseline. In contrast, the control arm demonstrated minimal to no change under the same conditions. This reduction indicates improved resistance of hair fibers to mechanical stress and enhanced structural integrity (Table 3).

**Table 3:** Change in hair parameters from before application to after application Summary of quantitative hair parameter assessments before and after application of the test and control products on standardized hair tresses. Data are presented as the number of samples (N), mean ± SD, and %CFB. The statistical significance of within-group changes from baseline was evaluated using a paired t-test and the Wilcoxon signed-rank test. Normality of the data was assessed using the Shapiro-Wilk test. SD: standard deviation, CI: confidence interval, %CFB: percentage change from baseline

Variables	Timepoints	Test product						Control product					
Statistics		N	Mean ± SD	%CFB	p-value	Test statistics	CI	N	Mean ± SD	%CFB	p-value	Test statistics	CI
Hair breakage assessment	Before application	6	30.67 ± 9.31	-	-	-	-	6	46.83 ± 12.32	-	-	-	-
(Dry fatigue)	After application	6	21.00 ± 6.07	-26.53%	0.0674	-2.33	(-20.34, 1.01)	6	51.50 ± 10.95	12.88%	0.2622	1.26	(-4.83, 14.16)
Hair breakage assessment	Before application	6	30.67 ± 9.31	-	-	-	-	6	46.83 ± 12.32	-	-	-	-
(Wet fatigue)	After application	6	18.00 ± 5.62	-36.86%	0.0292	-3.03	(-23.42, -1.91)	6	51.00 ± 4.69	16.22%	0.509	0.71	(-10.90, 19.23)
Hair tensile strength	Before application	6	20261.63 ± 7474.57	-	-	-	-	6	26033.60 ± 9168.06	-	-	-	-
	After application	6	25437.01 ± 6169.93	32.85%	<0.0001	10.06	(145.55, 6205.22)	6	23468.01 ± 7519.68	-7.79%	<0.0001	-4.57	(-3689.3, -1441.8)
Hair shine	Before application	6	1.52 ± 0.24	-	-	-	-	6	1.48 ± 0.14	-	-	-	-
	After application	6	2.15 ± 0.30	43.87%	<0.0001	-6.74	-	6	1.40 ± 0.17	-5.50%	<0.0001	-6.35	-
Hair volume	Before application	6	450.54 ± 15.72	-	-	-	-	6	450.64 ± 30.57	-	-	-	-
	After application	6	499.66 ± 34.30	10.84%	0.0042	4.96	(23.67, 74.58)	6	423.16 ± 15.81	-5.86%	0.045	-2.66	(-54.06, -0.91)
Split-end analysis	Before application	6	13.06 ± 2.60	-	-	-	-	6	12.25 ± 2.49	-	-	-	-
	After application	6	8.44 ± 2.88	-35.63%	0.0049	-4.79	(-7.10, -2.14)	6	12.34 ± 2.34	1.09%	0.6815	0.44	(-0.43, 0.61)
Length of 1^st^ strand	Before application	6	78.83 ± 18.81	-	-	-	-	6	77.71 ± 13.90	-	-	-	-
	After application	6	67.75 ± 21.71	-15.65%	0.003	-5.63	(-16.39, -5.77)	6	78.40 ± 14.57	0.78%	0.605	0.55	(-2.53, 3.91)
Length of 2^nd^ strand	Before application	6	82.35 ± 13.15	-	-	-	-	6	80.68 ± 15.45	-	-	-	-
	After application	6	64.16 ± 16.70	-22.84%	0.0044	-4.93	(-27.68, -8.71)	6	82.31 ± 15.43	2.39%	0.5411	0.66	(-4.79, 8.06)
Hair frizziness	Before application	6	128.62 ± 8.98	-	-	-	-	6	130.95 ± 7.83	-	-	-	-
	After application	6	89.20 ± 5.93	-30.37%	0.0003	-9.2	(-50.43, -28.40)	6	133.62 ± 8.56	2.03%	0.0404	2.75	(0.17, 5.15)
Hair root diameter	Before application	6	14.43 ± 2.85	-	-	-	-	6	16.51 ± 2.31	-	-	-	-
	After application	6	-	-	-	-	-	6	-	-	-	-	-
Hair shaft diameter	Before application	6	6.03 ± 1.13	-	-	-	-	6	6.39 ± 0.90	-	-	-	-
	After application	6	6.15 ± 1.16	2.01%	0.0008	7.15	(0.08, 0.17)	6	6.37 ± 0.90	-0.28%	0.2474	-1.31	(-0.05, 0.02)
Hair conditioning	Before application	6	1.5	-	-	-	-	6	1.33	-	-	-	-
	After application	6	8.33	-	-	-	-	6	1.33	-	-	-	-
Water uptake	Before application	6	46.84 ± 16.97	-	-	-	-	6	68.17 ± 9.15	-	-	-	-
	After application	6	84.03 ± 6.41	101.91%	0.0083	4.22	(14.53, 59.85)	6	38.69 ± 13.60	-43.36%	0.0019	-6	(-42.11, -16.83)
Water retention	Before application	6	60.56 ± 10.57	-	-	-	-	6	70.91 ± 9.57	-	-	-	-
	After application	6	69.08 ± 2.29	17.10%	0.0603	2.42	(-0.54, 17.57)	6	55.96 ± 9.20	-20.54%	0.0174	-3.49	(-25.95, -3.95)

Hair tensile strength

Following application of the test and control products, the test product arm showed an increase in hair tensile strength compared to the baseline. In contrast, the control arm demonstrated minimal to no change under the same conditions. This increase indicates improved mechanical strength of the hair fibers and may contribute to reduced susceptibility to breakage during routine grooming (Table 3).

Hair shine

Following the application of the test and control products, the test product arm showed increased hair shine compared to the baseline. In contrast, the control arm demonstrated minimal to no change under the same conditions (Table 3).

Hair volume

Following application of the test and control products, the test product arm showed an increase in hair volume compared to baseline. In contrast, the control arm demonstrated minimal to no change under the same conditions. This increase suggests improved surface smoothness and light reflectance in the hair fibers, contributing to a healthier, more lustrous appearance of the hair (Table 3).

Hair color

Following application of the test and control products, the test product arm showed a change in hair color parameters compared to baseline. In contrast, the control arm demonstrated minimal to no change under the same conditions. This change suggests improved color retention and stability of the hair fibers following repeated wash cycles (Table 4).

**Table 4:** Change in hair color parameter from before application to after application Mauchly’s test for assumption of sphericity; if the assumption of sphericity is violated, Greenhouse-Geisser corrections are used. Post hoc pairwise comparisons were adjusted using the Bonferroni correction. Summary of quantitative hair parameter assessments before and after application of the test and control products on standardized hair tresses. Data are presented as number of samples (N), mean ± SD, and %CFB. The statistical significance of within-group changes from baseline was evaluated using repeated-measures ANOVA. Mauchly’s test was used to assess the assumption of sphericity, and when this assumption was violated, Greenhouse-Geisser corrections were applied. Post hoc pairwise comparisons between visits were adjusted using Bonferroni correction. Normality of the data was assessed using the Shapiro-Wilk test. L* represents the lightness of the sample along the black-white axis, ranging from 0 (black) to 100 (white), where higher values indicate greater brightness. a* represents the position along the red-green axis, with positive values indicating redness and negative values indicating greenness, and is commonly associated with erythema. b* represents the position along the yellow-blue axis, with positive values indicating yellowness and negative values indicating blueness, and is often associated with pigmentation characteristics. SD: standard deviation, CI: confidence interval, %CFB: percentage change from baseline, ANOVA: analysis of variance

Variables	Timepoints	Test product								Control product							
Statistics		N	Mean ± SD	%CFB	Mauchy-test	p-value	p-value	Test statistics	CI	N	Mean ± SD	%CFB	Mauchy-test	p-value	p-value	Test statistics	CI
L*	Before application	6	15.52 ± 0.33	-	<0.001	0.0074	-	18.05	-	6	15.53 ± 0.30	-	<0.001	<0.001	-	51.54	-
	1^st^ wash	6	16.83 ± 0.62	8.56%			0.1270		(-2.97, 0.34)	6	16.88 ± 0.60	8.63%			0.011		(-2.29, -0.39)
	7^th^ wash	6	16.88 ± 0.63	8.89%			0.1128		(-3.04, 0.30)	6	16.92 ± 0.63	8.90%			0.0115		(-2.38, -0.39)
	15^th^ wash	6	17.01 ± 0.68	9.72%			0.0889		(-3.22, 0.22)	6	17.06 ± 0.66	9.81%			0.0102		(-2.59, -0.46)
	30^th^ wash	6	17.17 ± 0.61	10.71%			0.0410		(-3.23, -0.08)	6	17.23 ± 0.58	10.92%			0.0027		(-2.59, -0.81)
a*	Before application	6	4.15 ± 0.73	-	0.0306	<0.001	-	52.94	-	6	4.11 ± 0.36	-	0.1133	<0.001	-	98.39	-
	1^st^ wash	6	3.19 ± 0.62	-24.34%			0.0121		(0.28, 1.77)	6	3.04 ± 0.52	-28.86%			0.0031		(0.56, 1.88)
	7^th^ wash	6	2.23 ± 0.06	-45.44%			0.0104		(0.59, 3.36)	6	2.16 ± 0.03	-48.43%			<0.001		(1.31, 2.84)
	15^th^ wash	6	1.74 ± 0.32	-58.16%			0.0019		(1.27, 3.68)	6	1.61 ± 0.31	-61.98%			<0.001		(1.93, 3.34)
	30^th^ wash	6	1.50 ± 0.38	-65.10%			0.0033		(1.26, 4.31)	6	1.46 ± 0.38	-66.94%			<0.001		(1.75, 3.97)
b*	Before application	6	22.09 ± 1.76	-	0.0113	<0.001	-	34.36	-	6	22.09 ± 1.76	-	0.0494	<0.001	-	41.19	-
	1^st^ wash	6	20.42 ± 0.88	-7.32%			0.1557		(-0.55, 3.89)	6	19.95 ± 1.03	-9.44%			0.1361		(-0.6, 4.9)
	7^th^ wash	6	19.68 ± 1.12	-10.70%			0.0717		(-0.22, 5.05)	6	19.07 ± 0.91	-13.44%			0.0175		(0.65, 5.4)
	15^th^ wash	6	18.89 ± 1.29	-14.28%			0.0377		(0.21, 6.2)	6	18.16 ± 1.20	-17.53%			0.0207		(0.72, 7.14)
	30^th^ wash	6	17.63 ± 1.14	-19.97%			0.0088		(1.44, 7.48)	6	16.93 ± 0.99	-23.16%			0.0034		(2.32, 8.01)

Gravimetric analysis

Following application of the test and control products, the test product arm showed increased water uptake compared with baseline. In contrast, the control arm demonstrated minimal to no change under the same conditions. This increase indicates enhanced hydration capacity of the hair fibers and improved moisture absorption properties (Table 3).

Following application of the test and control products, the test product arm showed increased water retention compared to baseline. In contrast, the control arm demonstrated minimal to no change under the same conditions. This increase indicates an improved ability of the hair fibers to retain moisture and may contribute to better hydration and reduced dryness (Table 3).

Porosity analysis

Following application of the test and control products, the test product arm showed a shift in hair porosity from low to normal levels in the majority of hair tresses. In contrast, the control arm demonstrated no change under the same conditions. This shift indicates improved balance between moisture absorption and retention in hair fibers, contributing to better overall hair manageability (Table 3).

Split-end analysis (distance between two strands)

**Figure 3:**
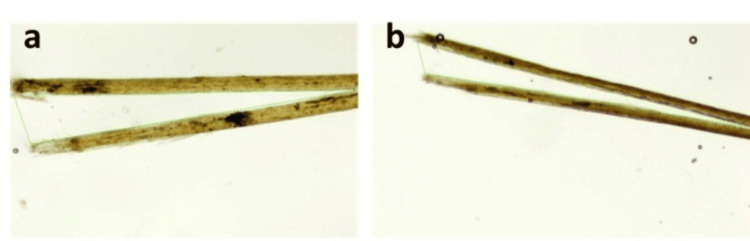
Change in split ends Microscopic images illustrating split-end analysis of hair strands. (a) Baseline image showing visible split ends with increased separation between hair fibers, and (b) post-application image showing reduced split-end separation after treatment with the test product.

Following application of the test and control products, the test product arm showed a reduction in the distance between split hair strands compared to baseline. In contrast, the control arm demonstrated minimal to no change under the same conditions. This reduction indicates improved alignment of the split fibers and may contribute to a smoother hair appearance with reduced visible damage (Figure 3).

Following application of the test and control products, the test product arm showed a reduction in the length of the first split strand compared to baseline. In contrast, the control arm demonstrated minimal to no change under the same conditions (Table 3).

Following application of the test and control products, the test product arm showed a reduction in the length of the second split strand compared to baseline. In contrast, the control arm demonstrated minimal to no change under the same conditions. This reduction suggests improvement in split-end condition and may contribute to reduced visible hair damage (Table 3).

Hair frizziness

**Figure 4:**
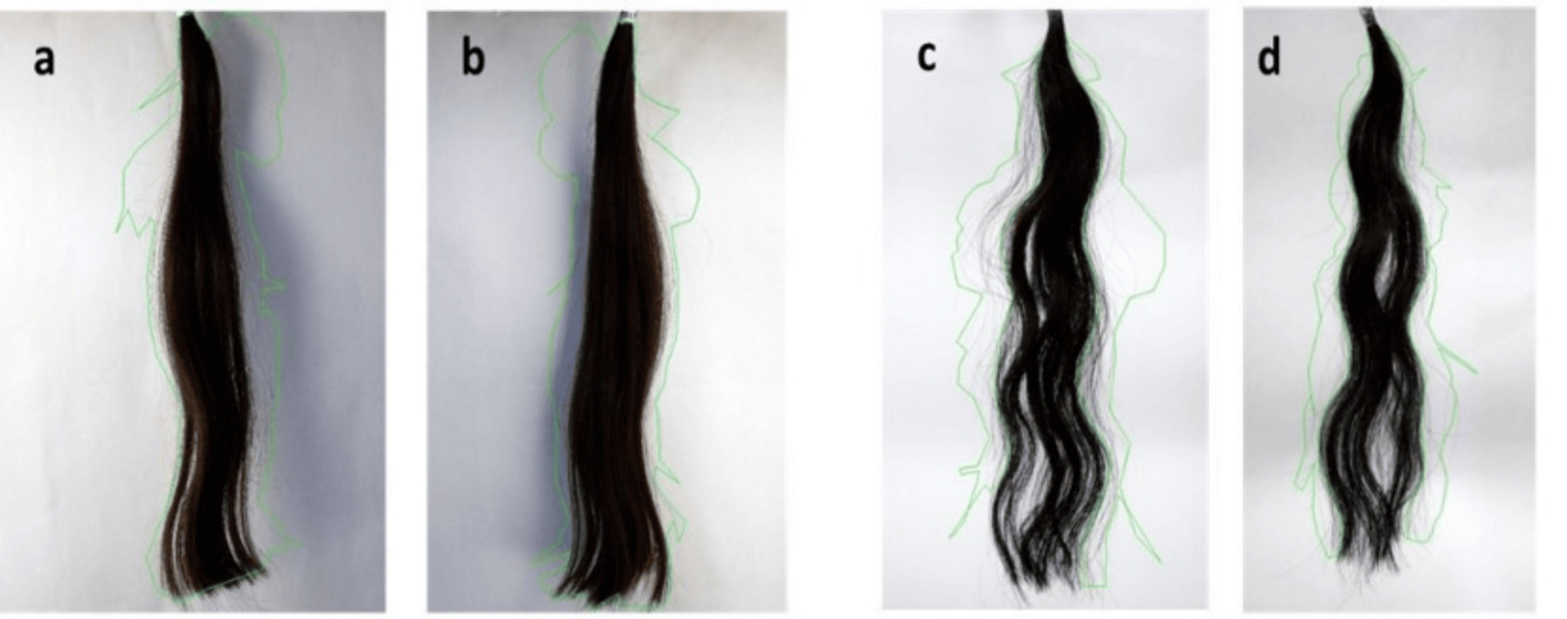
Change in hair volume and frizziness Representative images illustrating hair volume and frizz assessment using image-based analysis. The green outline indicates the quantified boundary of the hair tress used for analysis. (a) Baseline hair volume, (b) improved hair volume after application of the test product, (c) baseline hair frizz, and (d) reduction in hair frizz after application of the test product.

Following application of the test and control products, the test product arm showed reduced hair frizziness compared to baseline. In contrast, the control arm demonstrated no improvement under the same conditions. This reduction indicates improved alignment of hair fibers and may contribute to a smoother, more manageable hair appearance (Figure 4, Table 3).

## Discussion

In the current study, an attempt was made to establish a comprehensive in vitro test system to evaluate multifunctional hair claims by assessing various functional aspects of hair using standardized hair tresses in a laboratory setting. This work successfully incorporated several test methods, including sensory, mechanical, and optical analyses, microscopy, and imaging studies, to develop a scientific protocol for evaluating hair properties.

Several studies have used hair tress models because they provide a reliable experimental substrate. It is necessary that the standardized hair tresses must be obtained by ensuring that the hairs used originate from one source, are colored identically, have equal lengths, and are of similar weight. Similar methodologies have been reported by scientists for evaluating various mechanical and structural properties of hair in vitro [6].

In the current experiment, a systematic process of inducing damage using chemicals, heat, light, and environmental factors has been employed to mimic hair damage in real-life situations. Research has indicated that the use of surfactants, heat styling, and ultraviolet radiation may compromise the integrity of hair cuticles, increase porosity, and reduce the mechanical strength of hair strands.

The procedure also used several instrumental and image-based methods to objectively determine hair attributes. The use of tensile strength testers helped determine the breaking strength of hair fibers. In contrast, optical procedures such as glossymetry and colorimetry were used to objectively measure hair glossiness and color stability. Such instrumental approaches have been extensively employed in hair care science to characterize hair fiber attributes and the effectiveness of beauty products. Additionally, image analysis was used to assess key parameters, including split ends, hair frizz, and hair volume [7-10].

Additionally, the use of sensory tests provided a means to evaluate consumer-perceptible properties such as combing ability, feel, conditioning, and manageability. Sensory evaluation remains vital for assessing hair care products because it captures the changes consumers experience after use [11,12].

This study highlights the potential benefits of incorporating multiple standardized methods into a single experimental setting to develop a preliminary laboratory approach to assessing hair functionality. Additionally, the in vitro method proposed here provides controlled experimental assessments of hair performance.

On the other hand, since this study was carried out under laboratory conditions in vitro, using standardized hair tresses as test specimens, the results could be viewed only through the prism of the test substance's physical and chemical properties and their influence on hair fiber. They cannot be directly transferred into real-life consumer conditions. In vitro tests are well-suited for assessing mechanical strength, shine, frizz protection, and hair-structure maintenance; however, they do not involve biological processes such as hair growth or hormonal responses. The study has certain limitations. The experimental evaluations were performed using a relatively small number of standardized hair tresses under controlled laboratory conditions. The study evaluated isolated hair fibers only and therefore does not reflect biological processes such as hair growth, follicular activity, or scalp interactions. Additionally, advanced structural analytical techniques and placebo-controlled comparisons will be included in future studies.

## Conclusions

The present in vitro study proposes a preliminary, integrated hair-tress-based assessment framework for the systematic evaluation of multifunctional cosmetic hair care performance under controlled laboratory conditions. By incorporating sensory, mechanical, optical, and image-based assessment techniques, the methodology enables evaluation of multiple hair performance parameters, including hair breakage, tensile strength, shine, volume, frizz control, split-end appearance, and moisture-related properties. The incorporation of controlled damage-induction procedures further enhances the model's practical relevance by simulating common environmental and styling-related hair-stress conditions. The findings demonstrate that the selected assessment methodologies can detect measurable cosmetic changes and changes in hair properties following product application in standardized laboratory settings.
